# Polarization tunable all-dielectric color filters based on cross-shaped Si nanoantennas

**DOI:** 10.1038/s41598-017-07986-z

**Published:** 2017-08-14

**Authors:** Vishal Vashistha, Gayatri Vaidya, Pawel Gruszecki, Andriy E. Serebryannikov, Maciej Krawczyk

**Affiliations:** 1 Faculty of Physics, Adam Mickiewicz University in Poznan, Poznań, Poland; 2 Centre of Excellence in Nanoelectronics - CEN, IIT Bombay, Mumbai, India

## Abstract

Polarization sensitive and insensitive color filters have important applications in the area of nano-spectroscopy and CCD imaging applications. Metallic nanostructures provide an efficient way to design and engineer ultrathin color filters. These nanostructures have capability to split the white light into fundamental colors and enable color filters with ultrahigh resolution but their efficiency can be restricted due to high losses in metals especially at the visible wavelengths. In this work, we demonstrate all-dielectric color filters based on Si nanoantennas, which are sensitive to incident-wave polarization and, thus, tunable with the aid of polarization angle variation. Two different information can be encoded in two different polarization states in one nanostructure. The nanoantenna based pixels are highly efficient and can provide high quality of colors, in particular, due to low losses in Si at optical frequencies. We experimentally demonstrate that a variety of colors can be achieved by changing the physical size of the nonsymmetric cross-shaped nanoantennas. The proposed devices allow to cover an extended gamut of colors on CIE-1931 chromaticity diagram owing to the existence of high-quality resonances in Si nanoantennas. Significant tunability of the suggested color filters can be achieved by varying polarization angle in both transmission and reflection mode. Additional tunability can be obtained by switching between transmission and reflection modes.

## Introduction

Plasmonic nanostructures suggest a new platform for future printing technology, which promises to replace the old one that is based on organic dyes^1–4^. The normal organic dyes based inclusions can split the white light into fundamental color components, i.e., red, green and blue. However, the resolution of these dyes is not sufficient because of large thickness of the polymer that is needed to absorb a certain portion of the white light. Moreover, it has poor resolution which is degradable with time. In addition, these polymers are not eco-friendly. The new era in color filter technology is connected with the concept of light-matter interaction, that allows to engineer color filters having several important advantages over organic dyes based printing technology^5^. These advantages include ultra-small size, low power consumption, everlasting colors, and high-resolution wide color gamut even below the diffraction limit^6–9^. In fact, the first ideas regarding interaction of light with a nanostructure have been developed in the ancient time. In particular, the Roman Lycurgus Cup should be mentioned^10^. Simple examples of light-matter interaction can be observed in nature such as color of sky, wings of butterflies, beetles, and the feathers of peacocks^11, 12^.

The light-matter interaction depends on size and shape of the object. Generally, operation of a plasmonic color filter is based on separation of the white light components with the aid of the engineered plasmon resonance. The resonance characteristics can be statically tuned by altering the physical size of the nanostructure and its composition, which in turn determine wavelengths, at which light is predominantly scattered, absorbed, or transmitted^13^. But what is most interesting and important for practical applications is that these specific wavelengths also depend on the angle of incidence and polarization of incident light. These dependencies can be efficiently utilized in design of dynamically tunable color filters that do not need external bias. Several polarization sensitive color filters have earlier been reported, which operate either in transmission mode^14–19^ or reflection mode^9, 20–22^. Most of the known performances are realized using plasmonic (metal) nanostructures. Earlier, the choice for realization of these nanostructures was gold and silver. However, gold is costly for large scale fabrication. It also suffers of interband transitions in the visible frequency range, while silver faces the problem of aging, since it is highly reactive with native oxides^23^. Aluminum might be a good choice because of high stability and low cost^24–26^. Unfortunately, Al is even more lossy due to interband transition in visible range. Ultimately, plasmonic color filters have high losses at the visible wavelength regime. Most of them operate in transmission mode. Few of them operate in the dual mode that involves reflection and transmission modes^27, 28^. Recently, the investigations have been conducted with the aim to design and fabricate all-dielectric color filters^29–31^ and hybrid ones which combine metallic and dielectric components^15^. Another methodology is based on the use of complementary design methods with the hope of high-quality saturated colors^32^, which can cover a wide gamut on CIE-1931 chromaticity diagram. However, operation of the all-dielectric color filters suggested to the time is polarization insensitive.

All-dielectric, metasurface-based devices are considered as a promising alternative to metallic nanostructures due to significant advantages such as high-quality resonances and low intrinsic ohmic losses^33–40^. The advantages of Si nanodisks include high refractive index and ease of integration within the well established CMOS technology. The high refractive index of Si allows one to properly manipulate magnetic and electric components of light simultaneously, while in case of metal nanoantennas absorption losses are high and interaction with magnetic component of light is poor. Recently, all-dielectric structures based on Si nanoresonators have been suggested for creating low-loss high-quality color filters^29, 41^. All-dielectric color filters based on nanoantennas enable high quality of the colors with extended gamut^41^. These filters operate in reflection mode and show high quality of the colors on the chromaticity diagram. The high-quality saturated colors are enabled by Si nanoantennas due to the inherent property of high-quality resonances, i.e., sharper resonances than for metal nanoantennas. This results in wide gamut of saturated colors^41^. The recent progress in the area of light-matter interaction allows to efficiently utilize different spectral components of light in different regimes in one device.

In this work, we demonstrate all-dielectric, polarization sensitive color filters, which are composed of nonsymmetric Si nanoantennas, unlike the earlier reported ones that use metal based plasmonic nanoantennas^14–17^. It is demonstrated that the proposed color filters are strongly sensitive to the incident-wave polarization due to nonsymmetric cross shape of the used nanoantennas and, thus, they can be tuned by changing polarization. The nanoantennas show low losses at the visible spectrum and high confinement of light. We experimentally demonstrate that our color filters can operate in transmission and reflection modes, and can be tuned by changing the incident-wave polarization in each of these modes. The low losses of Si nanoantennas result in high quality of colors in both modes. The designed nanoantennas are nonsymmetric and cross-shaped so two information can be encoded in one physical structure, and then decoded by using two incident waves with orthogonal polarization states. In contrast with the recently reported color filters, the proposed device combines advantages of all-dielectric design and related wide gamut of highly saturated colors with advantages of sensitivity to polarization and dual mode operation.

## Results

### Operation Principles

**Figure 1:**
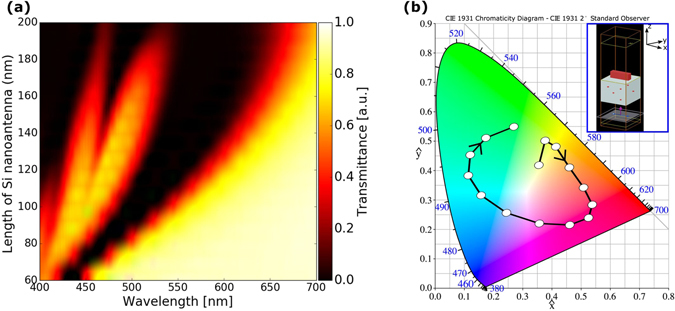
(**a**) Transmittance of Si rectangular nanoantennas on quartz substrate, when nanoantenna length is gradually varied from 60 nm to 200 nm. (**b**) Representation of transmittance spectra on CIE-1931 chromaticity diagram; blue numbers indicate wavelengths in nm for the ideal colors. Black line with circles indicates color changes when nanoantenna length is varied from 60 nm (first circle) to 200 nm (last circle); arrow shows direction in which length is increased. Inset shows unit cell of the simulated array of rectangular shaped Si nanoantennas with periodic boundary conditions; unit cell boundaries coincide with equicoordinate surfaces of Cartesian coordinate system.

First, let us consider an array of rectangular Si nanoantennas. It is excited by *x*-polarized plane wave in the visible range. The nanoantennas are shown to be scalable by means of the length variation. Width, length, and height have initially been chosen as 40 nm, 60 nm, and 200 nm, respectively, and then length varied from 60 nm to 200 nm. The simulated results for transmittance spectrum are presented in Fig. 1(a). A dip in transmittance spectrum for certain wavelength corresponds to certain color selectivity^17^. Thus, in order to obtain different colors for different nanoantenna lengths, it is necessary to have a transmission dip at the visible spectrum, and it must be shifted from lower visible spectrum to upper visible spectrum to achieve any arbitrary color.

**Figure 2:**
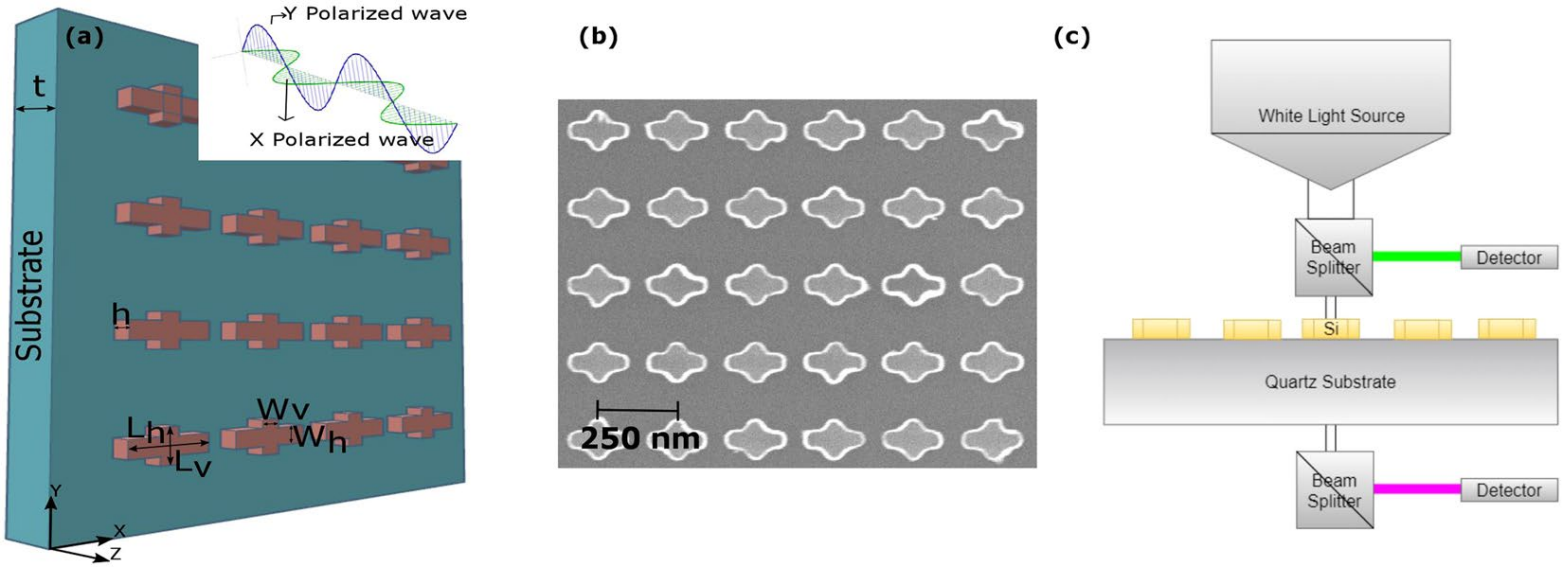
(**a**) Metasurface composed of nonsymmetric Si nanoantennas placed on top of quartz substrate; inset schematically shows wave propagation in case of *x*-polarized (Φ = 0°) and *y*-polarized (Φ = 90°) normally incident wave. (**b**) Top view of SEM image of fabricated device. (**c**) A schematic representation of the color filter operating in transmission and reflection modes.

As expected, the transmission dip occurs and is shifted towards larger wavelengths, while the nanoantenna length is increased. The extinction cross section of the corresponding single nanoantenna is also shifted towards larger wavelengths (see more details in supplementary information, Fig. S1). In Fig. 1(b), the obtained transmittance spectrum is converted into colors presented with the aid of CIE-1931 chromaticity diagram. One can see that different colors can be possible by partial scaling of rectangular nanoantennas that is achievable by varying their lengths. From the presented simulation results, it follows that an arbitrary color can be achieved by properly selecting the length. Using these results, we have selected two rectangular nanoantennas and combined them to form a nonsymmetric cross. Freedom in choice of the length ratio of the longer (horizontal) to the shorter (vertical) segment allows us to obtain significant difference in resonance frequency for these segments and make the resulting structure sensitive to polarization of the incident wave. Thus, operation of the cross-shaped nanoantennas and metasurface on their basis depends on polarization state. The general geometry of the designed metasurface is shown in Fig. 2. The lengths of the horizontal (*L*_*h*_) and vertical (*L*_*v*_) segments of each nonsymmetric nanoantenna are 150 nm and 90 nm, respectively, while the width (*W* = *W*_*h*_ = *W*_*v*_) and height (*h*) are 40 nm and 200 nm for the both segments. The nanoantennas form a square lattice with the lattice constant of 250 nm. The Si nanoantennas are placed on top of the quartz substrate, whose thickness is *t* = 275 ± 5 μm, see Fig. 2(a). A schematic illustrating the structure excitation by using the *x*-polarized (Φ = 0°) and the *y*-polarized (Φ = 90°) normally incident waves (Φ denotes the polarization angle in the (*x*,*y*)-plane) is shown in Fig. 2(a), inset. Figure 2(b) presents the SEM image of the fabricated structure. The schematic of the device operating in transmission and reflection mode is presented in Fig. 2(c).

Response at each of two orthogonal polarizations is mainly determined by the length of either the horizontal or the vertical segment. For an arbitrary polarized, normally incident wave, the combined response, i.e., total transmission due to both polarization components, is given by the following equation:

1 $$T({\rm{\Phi }},\lambda )={T}_{v}(\lambda )\,{\sin }^{2}\,{\rm{\Phi }}+{T}_{h}(\lambda )\,{\cos }^{2}\,{\rm{\Phi }},$$

where Φ is polarization angle and *λ* is free-space wavelength. *T*_*v*_(*λ*) and *T*_*h*_(*λ*) mean transmittance for *y*-polarized and *x*-polarized light, respectively.

**Figure 3:**
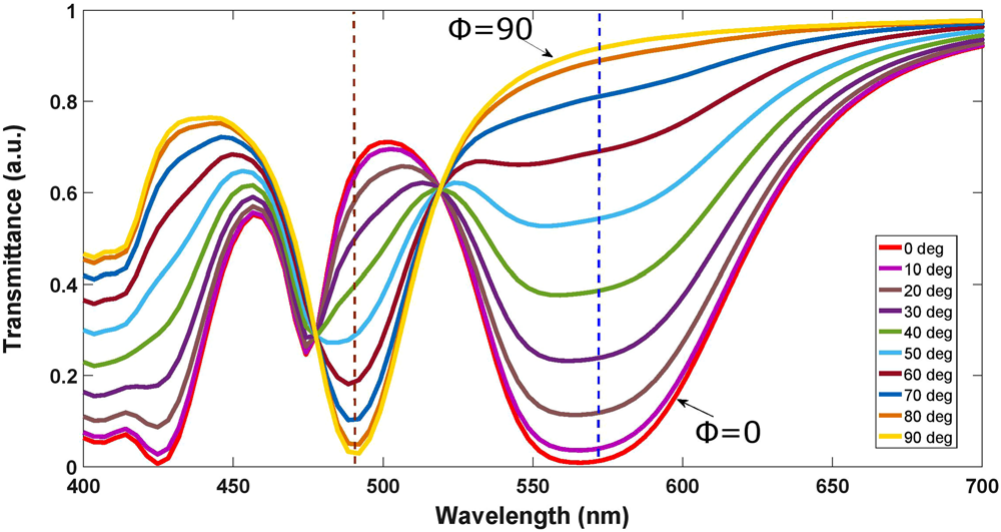
Shift in transmittance spectra when Φ is varied from 0° to 90°. Dashed lines indicate two resonance cases with strong transmission contrast between two orthogonal polarization states (Φ = 0° and Φ = 90°).

In order to illustrate sensitivity of the combined response of the device to variations of polarization, we performed simulations by varying Φ from 0° to 90° with the step of 10°. The results are presented in Fig. 3. One can see that strong redistribution of the incident-wave energy between the transmitted and reflected waves takes place in the whole wavelength range considered (see supplementary information, Figs S2–S5). Moreover, there is strong sensitivity to the polarization state. For instance, the lowest Mie resonance is expected to appear for the horizontal antenna segment near 570 nm, where the transmission dip appears for one of two orthogonal polarizations, i.e., at Φ = 0°, and then gradually disappears while increasing Φ. In turn, the transmission dip for the second orthogonal polarization, Φ = 90°, which should be connected with the lowest Mie resonance of the vertical segment, appears near 480 nm. The obtained results indicate that different colors can be obtained at different polarization states for a fixed set of geometrical parameters. As the length of the nanoantenna is increased, a higher Mie resonance appears for the horizontal segments, which leads to the additional dip of transmittance at 420 nm when Φ = 0°. More details regarding the effect of variation of Φ are given in supplementary information in Figs S2–S5. Two cases in Fig. 3 are interesting from the physics point of view, in which dependence on Φ tends to disappear. However, they are out of interest for the studied tunability mechanism which needs, in the contrast, strong sensitivity to variations in Φ.

**Figure 4:**
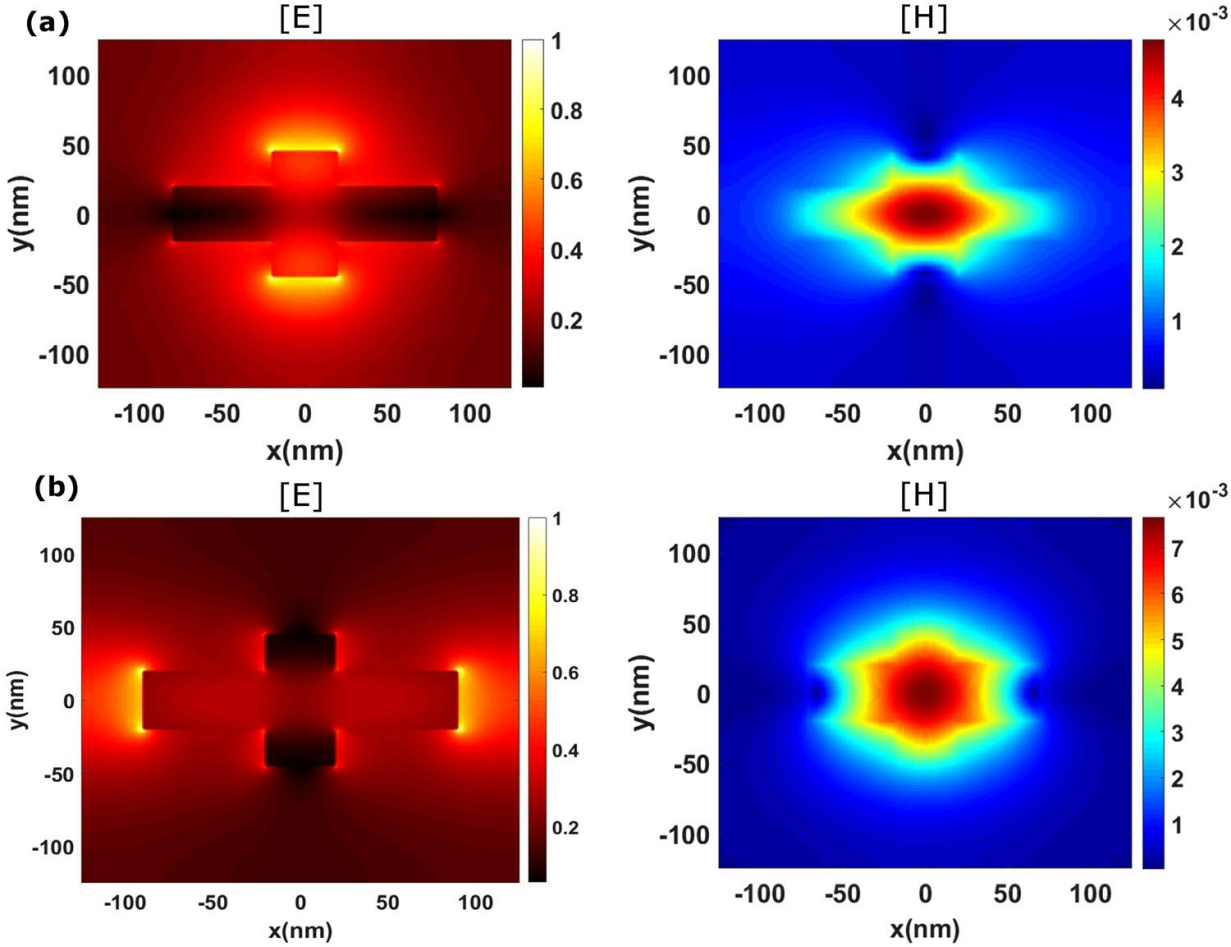
Electric and magnetic field distribution in (*x*, *y*)-plane, at the mid-height of nanoantenna (*z* = *h*/2): (**a**) *λ* = 480 nm, *y*-polarized incident wave and (**b**) *λ* = 570 nm, *x*-polarized incident wave.

To further clarify the operation principles of individual cross-shaped nanoantenna as a nanopixel, we have drawn the field distributions for two orthogonal states of polarization of the incoming wave. Each nanoantenna segment, i.e., vertical and horizontal one, responds individually when electric vector of incident wave align with the geometry. In Fig. 4, electric and magnetic fields are plotted for Φ = 0° and 90°. When the electric field vector of the incoming wave is align with horizontal or vertical nanoantenna segment, the corresponding segment behaves like an individual pixel, whose response can be tuned by varying the length. Thus, when Φ = 90°, only the vertical nanoantenna segment responds, that justifies the resonance observed in Fig. 3 near 480 nm. For Φ = 0°, the horizontal segment nanoantenna is in excitation mode, and this results in resonance observed in Fig. 3 near 570 nm. The magnetic field is confined around the center of the cross in both cases, so strong difference in electric field distribution is the origin of polarization sensitivity.

### Experimental Validation

Each spectral component of white light is associated with a certain color in transmission and reflection modes. We have selected and considered in detail two nanoantenna design cases with two different dimension sets which are given in Table 1. These sets are chosen so that we could demonstrate the primary colors, i.e., RGB or CMY. Indeed, we again observed different colors for different states of polarization of the incoming wave. In particular, RGB and CMY colors are obtained in reflection and transmission mode, respectively.

**Table 1:** Dimensions of the studied nanoantennas in nm.

	*L*_*v*_	*L*_*h*_	*W*_*v*_	*W*_*h*_	*h*
Case 1	90	160	40	40	200
Case 2	110	190	40	40	200

**Figure 5:**
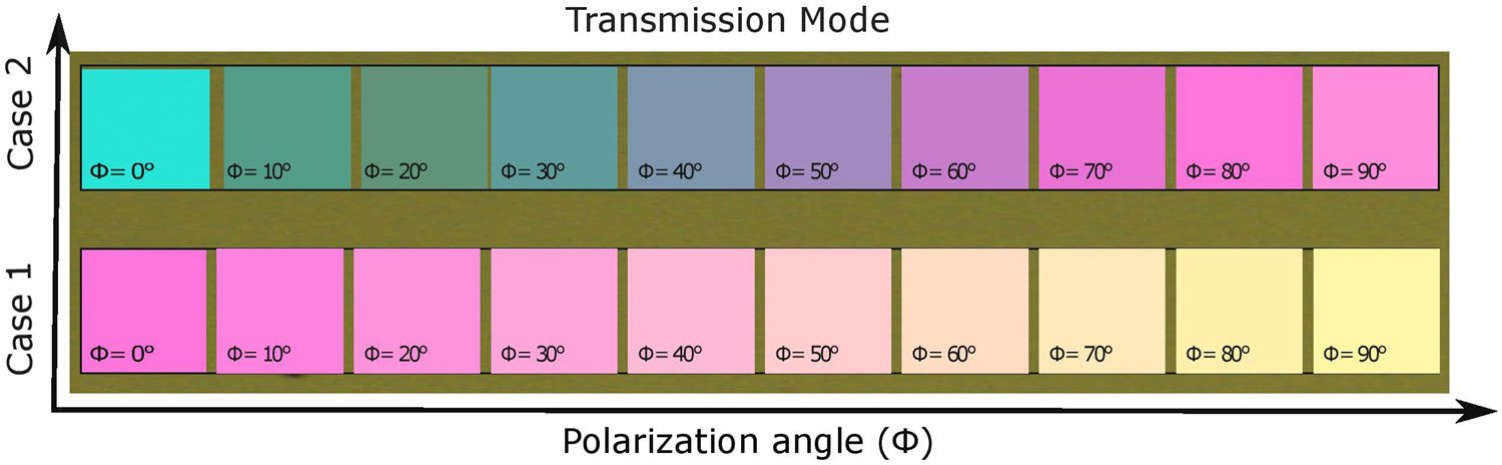
Colors visible under optical microscope in transmission mode when Φ is gradually varied from 0° to 90°. Dimensions of nanoantennas are given in Table 1.

Figure 5 presents the experimental colors observed under the optical microscope in transmission mode, when Φ is gradually varied from 0° to 90°. For Φ = 0°, color response corresponds to the excitation of horizontal (oriented along the *x*-axis) nanoantenna segments, whereas Φ = 90° corresponds to the excitation of vertical (oriented along the *y*-axis) nanoantenna segments. For given sizes, a variety of colors, each of which corresponds to a certain value of Φ, is obtained, so tunability can be realized by means of variations in Φ. The intermediate response between Φ = 0° to 90° can be understood from the above presented discussion for equation 1. The achievable set of colors is determined by the ratio of the lengths of the vertical and horizontal segments. Whilst location of the transmittance dip is a function of nanoantenna length, it can be shifted within a larger part of the visible spectrum by scaling nanoantennas. The effect of geometrical parameters of nanoantennas is illustrated by the comparison of Case 1 and Case 2 in Fig. 5. By properly adjusting the lengths of two segments of each cross-shaped Si nanoantenna, any two arbitrary colors can be formed using only Φ = 0° and Φ = 90° cases.

**Figure 6:**
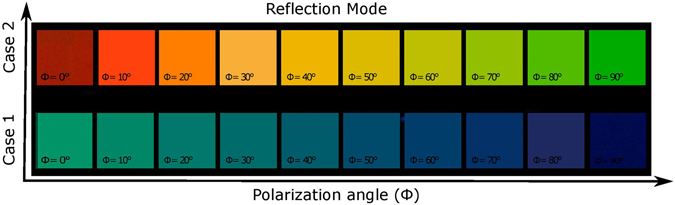
Colors visible under optical microscope in reflection mode when Φ is gradually varied from 0° to 90°. Dimensions of nanoantennas are given in Table 1.

A specific portion of the incident white light is reflected from the metasurface, while another part is transmitted through the array on quartz substrate, as schematically shown in Fig. 2(c). The dielectric nanoantennas have very low losses that enables creation of various high-quality colors in both transmission and reflection mode. When the same devices operate in reflection mode, we again obtain different colors for different states of polarization of the incoming wave. Figure 6 presents the experimental colors seen under the optical microscope in reflection mode when Φ is varied from 0° to 90°. As expected, the obtained set of colors is different than for transmission-mode operation. Again, the set of colors that are tunable by varying Φ depends on values of *L*_*v*_ and *L*_*h*_, so the difference in colors achievable in reflection mode in Case 1 and Case 2 is significant. Moreover, since different color sets are obtained in transmission and reflection modes, additional degree of freedom in tunability can be added by switching between these two modes.

**Figure 7:**
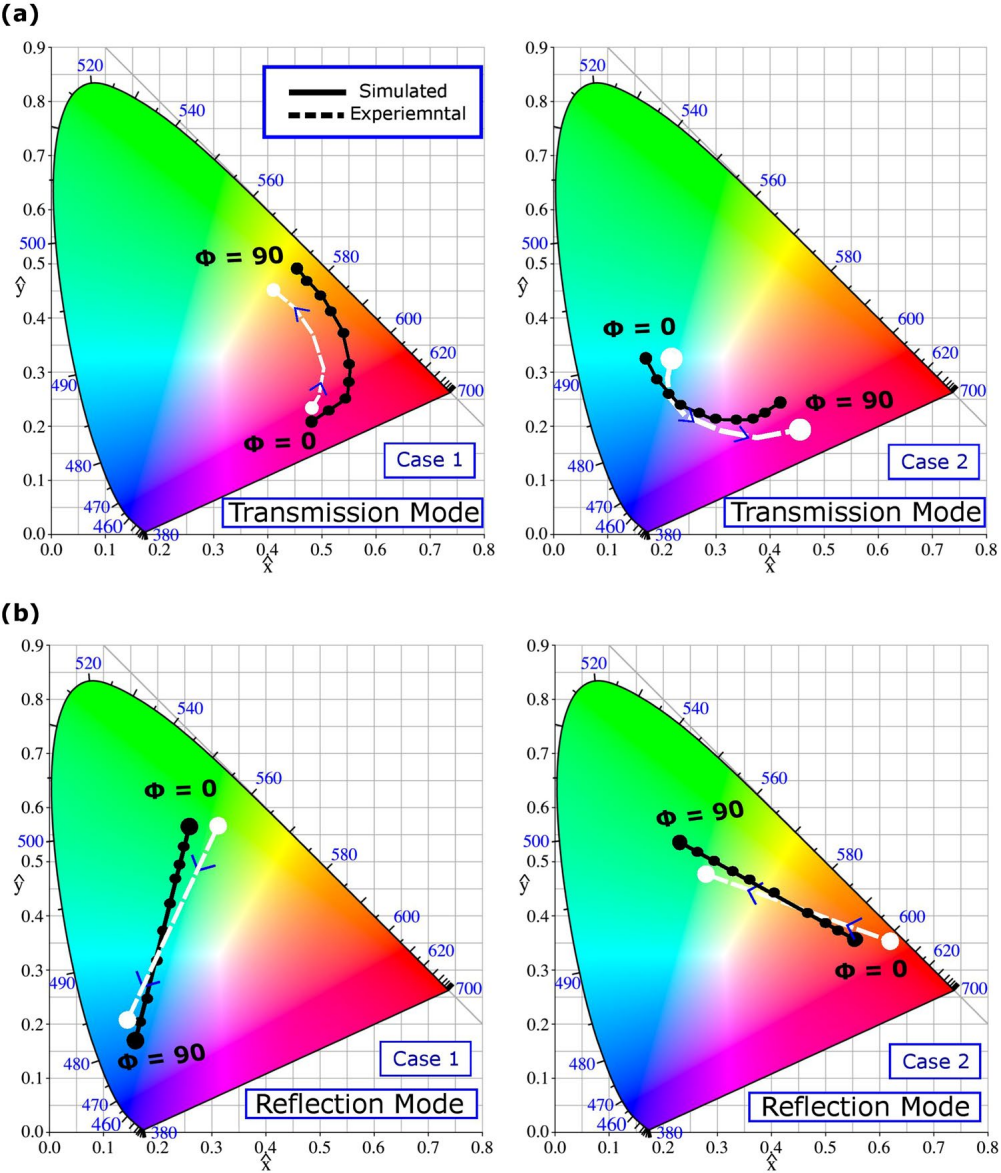
Polarization related changes in color: (**a**) for transmission mode, from magenta to yellow in Case 1 and from cyan to magenta in Case 2. (**b**) For reflection mode, from green to blue in Case 1 and from red to green in Case 2. Polarization angle of incident white light (Φ) is switched for experimental results from 0° to 90°; simulation results for polarization states, which correspond to Φ varied from 0° to 90° with the step ΔΦ = 10°, are shown for comparison. Blue numbers indicate wavelengths in nm for the ideal colors.

A dual characterization is carried out to justify the above discussed results. We have used a home-made customized setup for this purpose. The details of the optical characterization are available in supplementary information. The experimental transmittance and reflectance spectra are converted into the colors by using standard chromaticity matching functions^42^, see supplementary information. For Case 1 and Case 2, the gradual color changes are observed when polarization angle is gradually varied from 0° to 90°. In Fig. 7, the experimental and simulated results are plotted on the standard CIE 1931 chromaticity diagram, in order to visualize the achievable color tunability. Generally, these results are in good coincidence. Some discrepancy appears due to imperfections in fabrication and limitations of measurement accuracy. In particular, its possible reasons can be connected with that it can be difficult to fabricate a nanostructure with a high-aspect ratio. For Case 1 in transmission mode, it is expected from the simulation results that the changes in color should occur from magenta to yellow zone, when polarization of incident light is changed from 0° to 90°, and so happens in the experimental results with acceptable deviation. For Case 1 in reflection mode, color changes are predicted from green to blue zone, based on the simulation results, and so happens in the experimental results, also with acceptable deviation. A similar level of results coincidence has been found for Case 2, in both reflection and transmission modes. Hence, there is a good agreement between the simulation and experimental results, as far as the latter are still in the predicted zone of the colors. Further study of the effect of geometrical parameters of the cross-shaped nanoantennas and their optimization can be promising for obtaining alternative tunability scenarios and possible extension of the tuning range.

## Discussion

In summary, we have studied all-dielectric metasurfaces based on nonsymmetric cross-shaped Si nanoantennas, which are designed to operate as color filters in transmission and reflection modes. The proposed designs of low-loss all-dielectric filters enable high quality of colors for the both modes. The resonance location responsible for color selectivity can be varied in a desired way by properly adjusting sizes of individual nanoantennas. The nonsymmetric cross shape of nanoantennas makes them strongly sensitive to the polarization state of incident wave. Hence, efficient tuning of colors can be achieved in one structure just by changing polarization angle, as expected. The range of achievable colors depends on sizes of rectangular segments creating a cross. Tuning based on polarization change can be efficiently used in both transmission and reflection modes. In turn, switching between transmission and reflection modes gives more freedom for tuning. The proposed devices allow covering a wide gamut of colors on CIE-1931 chromaticity diagram. In particular, RGB or CMY colors are obtained in reflection and transmission mode, respectively. There is good coincidence between simulation and experimental results. Results of higher accuracy could be obtained by improvement of fabrication quality and measurement setup. The suggested devices have potential applications in the area of secured optical tag, nano spectroscopy, fluorescence microscopy and CCD imaging.

## Methods

### Simulations

We have used Lumerical FDTD^43^ solver to study transmission and reflection for the metasurfaces comprising cross-shaped nanoantennas on quartz substrate. The materials used for substrate and cross-shaped nanoantenna are SiO_2_ and Si, respectively. The material parameters are taken from the default material library of the used software. A plane wave ranging the wavelength from 400 nm to 700 nm is incident from the top of the structure. The reflectance and transmittance spectra are simulated by considering a unit cell (single cross-shaped nanoantenna on substrate) with periodic boundary conditions in *x* and *y* directions. Perfect matching layer (PML) boundary conditions were used in *z* direction to avoid reflections.

### Device fabrication

A piranha cleaned quartz sample (275 μm thick) is used to fabricate the device. We have deposited a thin layer of 200 nm amorphous Si using ICPCVD tool at 300 °C with 150 W added microwave power. A single-layer PMMA photoresist is used for patterning cross-shaped nanoantennas by using Raith 150-Two EBL tool. An electronic mask is designed using an open source Python program. The exposed sample is developed using MIBK-IPA (1:3) and an IPA solution for 45s and 15s, respectively. A thin layer of metal (5 nm Cr as adhesion layer and 40 nm Au) is deposited to transfer the pattern on metal layer for lift-off process using four target evaporators. After lift-off, the sample is etched using plasma asher to get the final pattern. A process flow chart with step by step details is available in supplementary information.

### Optical characterization

A dual optical characterization is done to ensure the results. The sample is placed under Olympus optical microscope and illuminated with white light without filter. The colors can be directly seen under optical microscope in reflection and transmission mode by changing the polarization of the incident light. The reflectance and transmission spectra are measured using a home-made customized setup. A HL 2000 halogen lamp source is coupled with optical fiber to illuminate the sample with the light in visible range (wavelength of 400 nm to 700 nm). A polarizer is added in the path of the optical fiber to control the polarization. A 50× objective lens is used to tight focus the light on the sample. The spectra are measured by using the same objective lens. The data are normalized with respect to bare quartz sample. A Nikon camera attached with the assembly is used to take the photograph of the illuminated area.

## Electronic supplementary material

Supplementary information
