# Supplementary material for: Polarization tunable all-dielectric color filters based on cross-shaped Si nanoantennas
Source: Sci Rep. 2017 Aug 14;7:8092. doi: 10.1038/s41598-017-07986-z (PMC5556121; doi:10.1038/s41598-017-07986-z)
Supplement: Supplementary file 1 — Supplementary information [file 41598_2017_7986_MOESM1_ESM.pdf]

Supplementary Information
Polarization tunable all-dielectric color filters based on cross-shaped Si nanoantennas

Vishal Vashistha,*,† Gayatri Vaidya,‡ Pawel Gruszecki,† Andriy E. Serebryannikov,† and Maciej Krawczyk*,†

†*Faculty of Physics, Adam Mickiewicz University in Poznan, Poland*

‡*Centre of Excellence in Nanoelectronics - CEN, IIT Bombay, India*

E-mail: visvas@amu.edu.pl; krawczyk@amu.edu.pl

Simulations Methodology

A commercially available Lumerical FDTD solver is used to simulate a single rectangular nanoantenna and nanoantenna arrays. Si and SiO_2 are used as materials for nanoantennas and substrate, respectively. Their parameters are taken from the material library of the used software. First, the extinction cross section (ECS) of a single rectangular dielectric nanoantenna has been calculated by using a total-field scattered-field (TFSF) source with the bandwidth adjusted to visible wavelength which extends from 400nm to 700nm. The perfectly matched layer (PML) boundary conditions are employed in all three orthogonal directions, i.e., x, y, and z. Fig. S1 shows the ECS of the nanoantenna, when length of the rectangular nanoantenna is changed from 60nm to 200nm.

At smaller length values, the scattering peak is shifted from lower to higher visible spectrum. When the length is increased, the peak is split into two peaks. The larger the length,

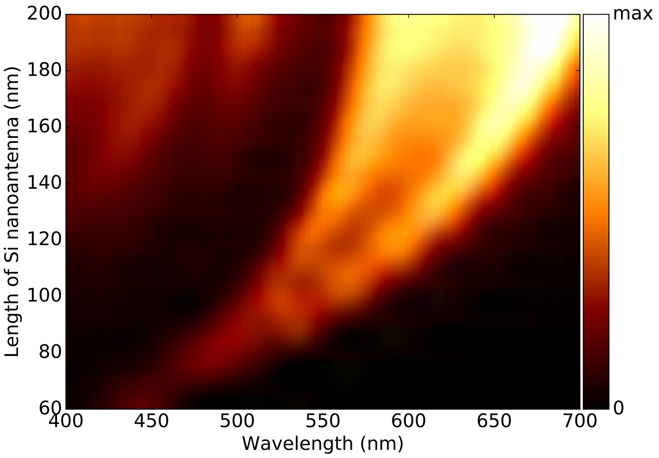

Figure S1: Extinction cross section (ECS) of single rectangular Si nanoantenna with width and height of 40nm and 200nm, respectively; its length is gradually increased from 60nm to 200nm. Two resonance peaks are associated with electric type and magnetic type resonances.

the stronger the peaks are shifted towards larger wavelengths. These two peaks can be associated with electric and magnetic resonances, which are known for dielectric nanoparticles. Some additional modes may also appear when the length of Si nanoantennas increases. However, the main contribution to scattering is provided by one or two modes, which manifest themselves in the shift of the ECS maxima from left to right, while the length of the Si nanoantennas is increased.

In order to get the reflectance and transmittance spectra for the array of cross-shaped nanoantennas, we have illuminated the metesurfaces with plane waves having different polarization states. We have used periodic boundary conditions in x and y directions and PML boundary conditions in z direction. In Figure S2, the color map of transmittance spectra is presented in wavelength - polarization angle (Φ) plane. Figure S3 illustrates the shift of the transmittance minima when Φ is switched from $0°$ to $90°$. Figures S4 and S5 present reflectance in the same manner and in the same ranges of parameter variations as Figs. S2 and S3.

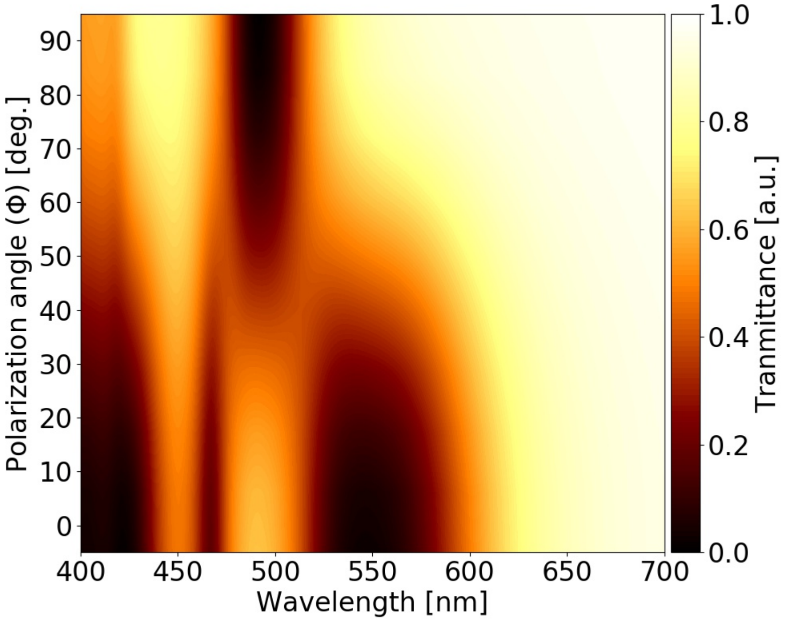

Figure S2: Color map of transmittance for arbitrary polarization state of incident wave in the visible region.

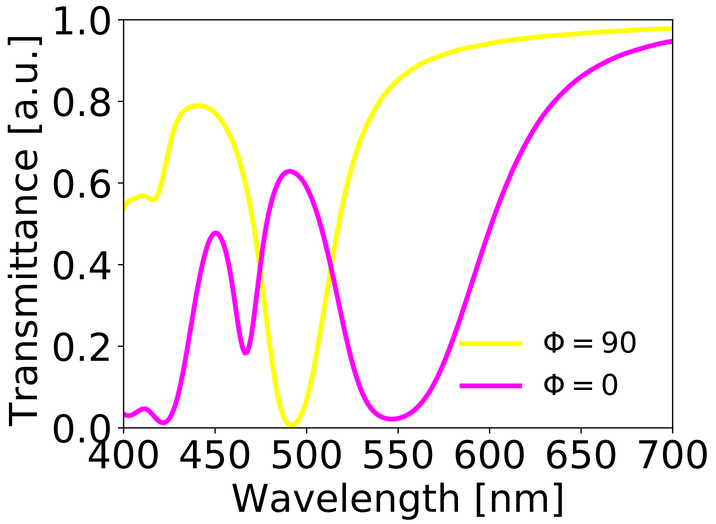

Figure S3: Transmittance at $\Phi = 0°$ and $\Phi = 90°$. Shift of the dip is clearly seen.

Color representation on CIE-1931 Chart

Color is the interesting phenomenon known from the centuries. A white light coming from the sun contains all the color components. There are many methods to classify the different colors in different categories based on the properties. Among them, classification based

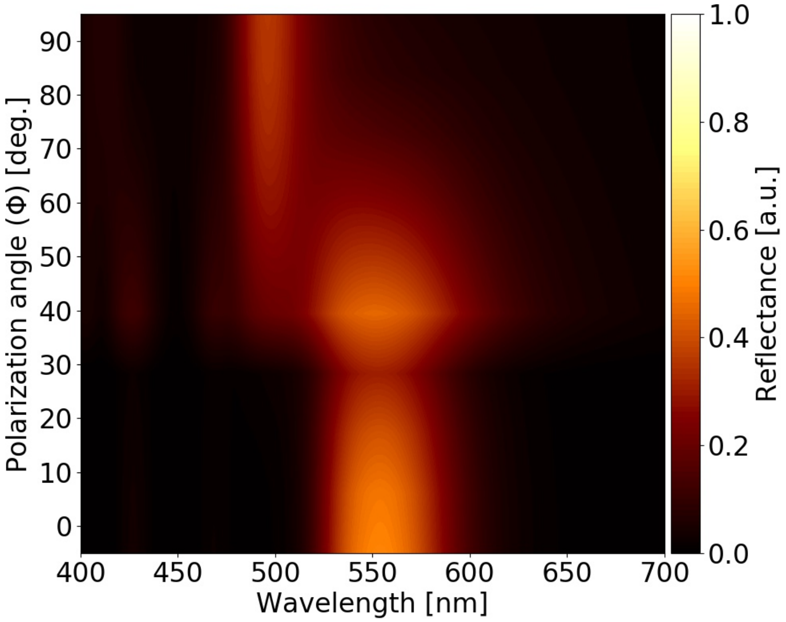

Figure S4: Color map of reflectance for arbitrary polarization state of incident wave, in the whole visible region.

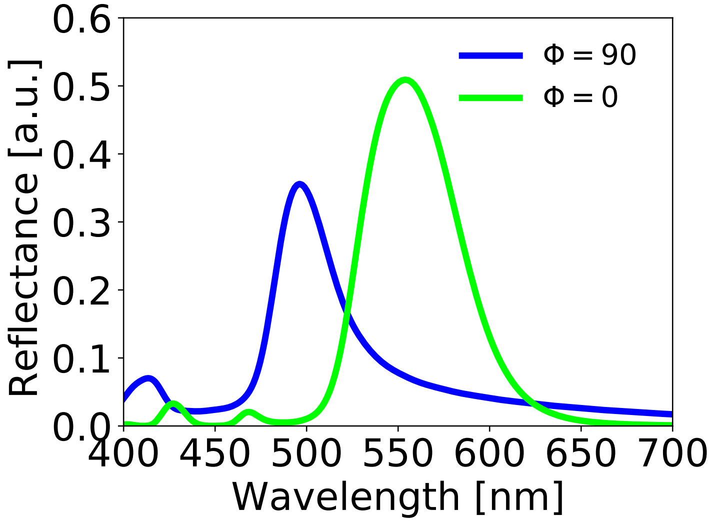

Figure S5: Reflectance at $\Phi = 0°$ and $\Phi = 90°$. Shift of the maximum is clearly seen.

on primary colors (RGB) is the most general one. When a white light is incident on the surface, reflection can be dominant at certain wavelengths or wavelength ranges, which are determined by surface properties. These wavelengths are perceived by human brain based on human reception system. The response is not the same for all wavelengths. The difference

can be taken into account by using the standard chromaticity matching functions. The resulting response functions were defined by Commission Internationale de l'Eclairage (CIE) as follows:

$$X = \int I(\lambda) * CIEX(\lambda) * d(\lambda), \tag{S1a}$$

$$Y = \int I(\lambda) * CIEY(\lambda) * d(\lambda), \tag{S1b}$$

$$Z = \int I(\lambda) * CIEZ(\lambda) * d(\lambda), \tag{S1c}$$

where $I(\lambda)$ is light intensity vs. wavelength and $CIEX(\lambda)$, $CIEY(\lambda)$, $CIEZ(\lambda)$ are the matching functions. All these chromaticity functions are defined over the interval of 360nm to 830nm, and are zero for all wavelengths outside this interval. These functions are plotted in Fig. S6. They all are positive everywhere within the interval, since the intensity of the light cannot be negative.

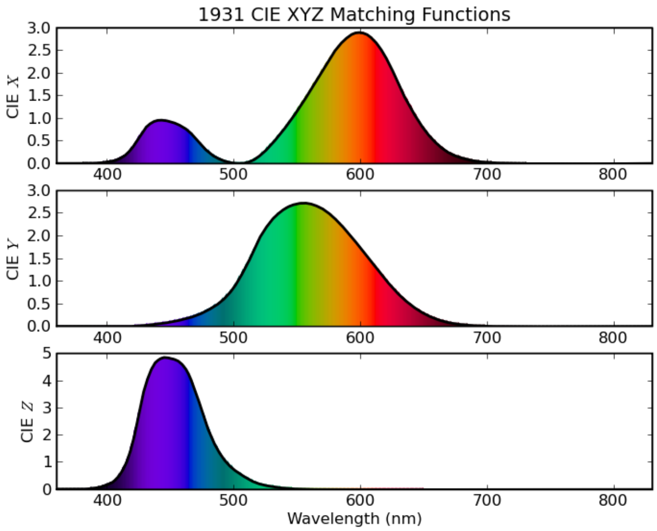

Figure S6: CIE chromaticity matching functions.

These chromaticity functions X, Y, and Z are normalized to new scale from 0 to 1. The

normalized functions are denoted by $\hat{x}$, $\hat{y}$, and $\hat{z}$. The normalization is done as follows:

$$\hat{x} = X/(X + Y + Z), \tag{S2a}$$

$$\hat{y} = Y/(X + Y + Z), \tag{S2b}$$

$$\hat{z} = 1 - \hat{x} - \hat{y}. \tag{S2c}$$

All possible colors are represented in $(\hat{x}, \hat{y})$-plane of CIE-1931 chromaticity diagram.

Device fabrication

A quartz sample (275µm thick) is used as substrate for the fabricated nanoantenna arrays. A standard piranha cleaning process is carried out. We have deposited a thin layer of 200nm amorphous Si using ICPCVD tool at 300°C. We used 150W microwave power, in addition to inductive coupled power, in presence of 10sccm SiH_4, 20sccm H_2, and 20sccm Ar. The process is carried out at very low pressure to get the high-quality deposition. A profilometer is used to confirm the thickness, and SEM imaging is carried out to check the quality of deposited sample. A single-layer positive photo-resist (PMMA 2%) is used for pattering the crosses at 4000 RPM for 45s. The pattern is transferred to the PMMA positive photo-resist by Raith 150 electron beam lithography (EBL) tool. This sample is developed using MIBK-IPA (1:3) and IPA solutions for 45s and 15s, respectively. In order to transfer this pattern on metal, we have deposited 5nm Cr layer (to increase adhesion) and 40nm Au layer on top of the patterned sample. These depositions are carried out with the aid of thermal evaporator. After depositing the metal, we have kept the sample in acetone for 15h to lift-off metal from the unwanted area. Finally, we have checked the pattern at the optical microscope to confirm the result of lift-off process. This sample is etched using a plasma etcher, where metal layer behaves like a hard mask to protect the patterned area. We used 30sccm ChF_3 and 10sccm O_2 to etch the amorphous Si anisotropically. The process was lasted for 4min. Finally, an

Au etchant and a Cr etchant are used to remove the metal mask layer. As a result, we obtain the array of Si cross shapes on top of the quartz substrate. A complete process flow is shown in Fig. S7.

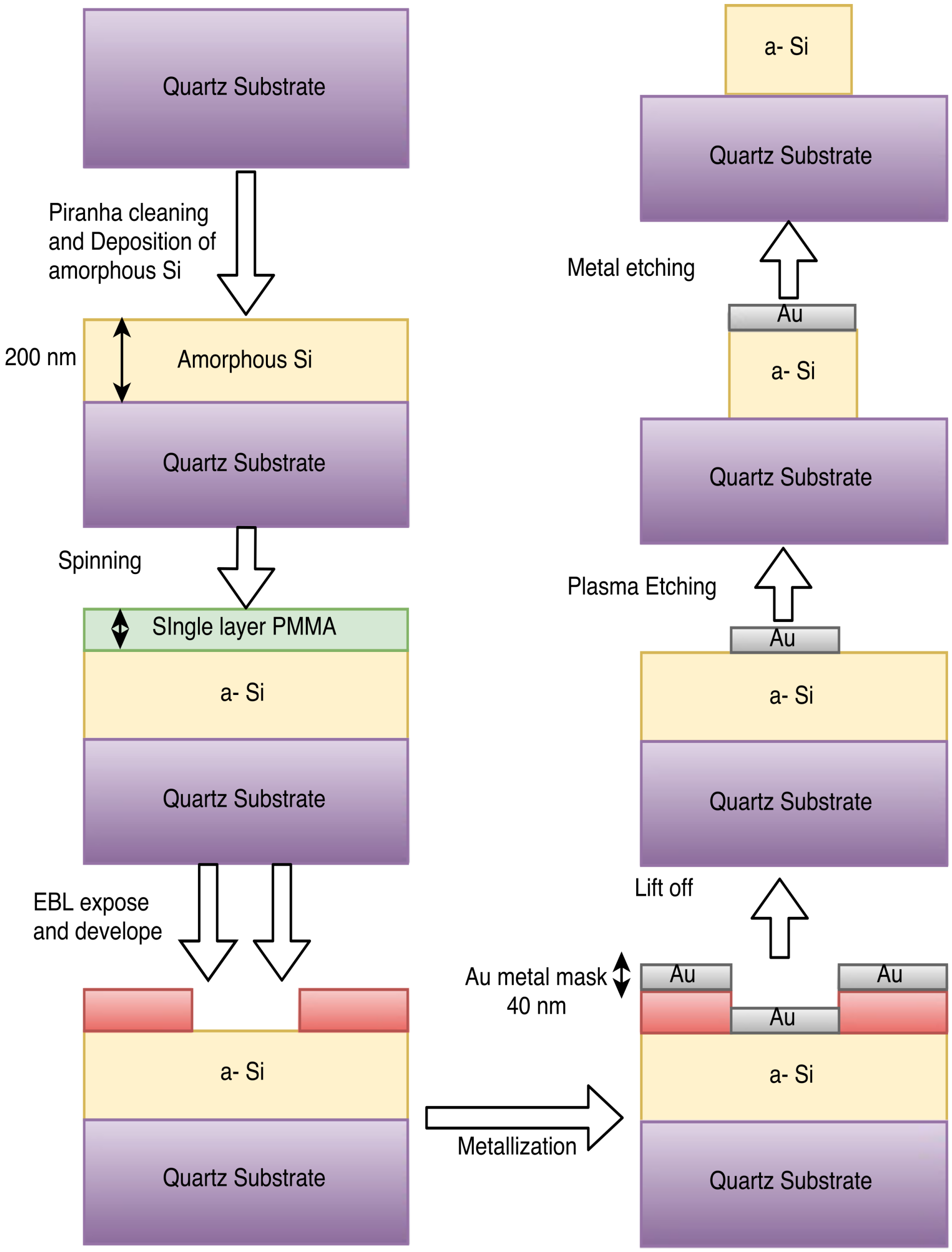

Figure S7: Process flow chart for fabrication.

Optical characterization

A dual optical characterization is done to ensure the correctness of the results. The sample is first placed under Olympus optical microscope illuminated with a white light without filter. The colors can be directly seen under optical microscope in reflection and transmission modes at various polarization angles. Then, reflectance and transmittance spectra are measured using a home-made customized setup. A HL 2000 halogen lamp source is coupled with optical fiber to illuminate the sample in the visible range, i.e., from 400nm to 700nm). A polarizer is added in the path of the optical fibre to control the polarization. A $50\times$ objective lens is used to focus the light on the sample. The reflectance and transmittance spectra are measured by using the same objective lens. The data are normalized with respect to the bare quartz sample. A Nikon camera attached with the assembly is used to take the photograph of the illuminated area.
